# Permanent resident

**DOI:** 10.3402/meo.v21.31160

**Published:** 2016-05-17

**Authors:** John F. Fisher

**Affiliations:** Medical College of Georgia, Augusta University, Augusta, GA, USA

**Keywords:** medical education, student and resident training, attending physician responsibilities

## Abstract

The training of physicians in the past century was based primarily on responsibility and the chain-of-command. Those with the bulk of that responsibility in the fields of pediatrics and internal medicine were residents. Residents trained the medical students and supervised them carefully in caring for patients. Most attending physicians supervised their teams at arm's length, primarily serving as teachers of the finer points of diagnosis and treatment during set periods of the day or week with a perfunctory signature on write-ups or progress notes. Residents endeavored to protect the attending physician from being heavily involved unless they were unsure about a clinical problem. Before contacting the attending physician, a more senior resident would be called. Responsibility was the ultimate teacher. The introduction of diagnosis-related groups by the federal government dramatically changed the health care delivery system, placing greater emphasis on attending physician visibility in the medical record, ultimately resulting in more attending physician involvement in day-to-day care of patients in academic institutions. Without specified content in attending notes, hospital revenues would decline. Although always in charge technically, attending physicians increasingly have assumed the role once dominated by the resident. Using biographical experiences of more than 40 years, the author acknowledges and praises the educational role of responsibility in his own training and laments its declining role in today's students and house staff.

Responsibility has always been my best teacher and she began her tutorial in January of 1968 on my first clinical rotation as a third-year medical student at the Medical College of Virginia (MCV) of Virginia Commonwealth University – Medicine at the McGuire VA Medical Center in Richmond, VA. I didn’t yet know how to draw blood or start an i.v., but I was assigned to be the ‘doctor’ for five patients my first morning on the ward. I say ward because that's what it was: an open ward of what must have been 20 patients. The two interns who were on my team were assigned the rest of the patients and the medicine resident supervised them and ‘defended’ my five from me. Some of these veterans certainly deserved an additional purple heart aside from the ones they were awarded in WW I for the multiple battle wounds I inflicted on their upper extremities prior to discharge. I don't doubt that many of the patients stuck with (and by) me as their doctor suffered post-traumatic stress disorder or battle fatigue as it was called in those days.

Nonetheless, on attending rounds, when we got to my patients, I alone had to report on the events of the past 24 hours and the pertinent physical findings and live or die by what I said. The attendings, two of them, patiently suffered through my disorganized and diffuse presentations and verified my amateurish physical exam with seasoned skill and aplomb – and skepticism. On one instance, they were quick to ask me if I had noticed the ghostly-white mucosal pallor and heard the loud S4 in one of my hypertensive, very dark, African-American patients. Only moments before, I had proudly proclaimed my success in bringing his blood pressure under good control with methyldopa. Triumph became despair in an instant. At this early stage in my career, my physical exam skills were only sufficient to confidently state that the patient's heart was present. The association of a fourth heart sound with hypertension I had read about in sophomore year, but William Osler re-incarnated I was not. My heart sank further as they flipped to the lab section of the bedside, aluminum-bound chart. I had overlooked his hemoglobin of 5 gm/dL in the admission labs. Thankfully, my resident had already ordered two units of blood for him before rounds unbeknownst to me. He ‘had the patients’ backs’, but not mine. I died that day on rounds and I came back to haunt the team many more times, but responsibility was quickly making inroads in my education. Happily, by the end of that 3-month rotation my supervisors noticed it.

My next rotation was a month-long experience in general medicine at a community hospital on the eastern shore of Virginia. There were two bright and charismatic internists supervising me and my student partner. We had 2 weeks with each physician, working up all new patients and making morning rounds, presenting all the events of the previous day. I could sense that my presentations were just barely beginning to sound like those of the house staff at the VA the previous 3 months. We were on-call every other night. Especially at night or whenever our supervising doctors were not in the hospital or over in their private offices a few blocks away, the nurses on any floor would call us first about any patient issue. Both of our supervisors expected us to examine the patient, decide what to do, and call them only if we were lost. We were their ‘residents’ and the ward clerks and nurses carried out our written and verbal orders and the two physicians countersigned them at some time before the patients’ discharges. Consequently, I quickly got pretty confident in ordering MOM 30 cc qhs prn; chloral hydrate 500 mg po qhs prn; 2 L of nasal O_2_; clear-liquid and 2-g sodium diets; chest radiographs; and CBCs, electrolytes, and blood glucoses; EKGs; and sputum and urine cultures. The pages of the PDR and Lange's *Current Medical Diagnosis and Treatment* of 1968 became ‘dog-eared’ from my constant looking things up to avoid hurting a patient or giving my attendings any idea I couldn't handle the pressure or was inept. For much of this ‘residency’ I didn't need to pester them about minor issues, but I knew my limits and called on them if I felt I had arrived at my own level of incompetence, which was daily. Another valuable lesson was being learned – recognizing when I needed help. Once again, responsibility was shaping me from what had begun 3 months ago as a hapless figure in a short, white coat into someone just beginning to have a semblance of reasonable judgment at the bedside of a sick patient.

The OB-GYN clinical clerkship at St. Philip Hospital on the MCV campus was the responsibility rotation of a lifetime. My roommate, life-long friend, and the person with the surname just ahead of mine on the class roster, Pasquale Finelli, and I shared every-other-night call in Labor and Delivery. There were so many mothers in active labor every night that the house officers could not possibly deliver all of their babies without the help of Pat and me. I am forever grateful for the saintly patience of the L & D nurses as they talked me through each step of my first few deliveries. They had no other choice – the resident and interns were busy with problems of their own in other rooms with less straight-forward cases. There was a lot of hollering on that floor from primigravidas in pain and anxiety, from hurried doctors under stress, and from nurses scrutinizing the women who were effaced and dilating hoping to alert the resident the name and bed location of the woman who needed to be delivered next and trying to avoid a precipitous ‘visit from the stork’ in the bed. Cat-napping on a gurney at 3:00 AM was typically the only sleep we got and was especially likely to be interrupted by a call from a nurse like, ‘Gravida nine ready to go’. Many of these experienced mothers could have delivered their own baby, but they were inexplicably re-assured by my presence in gown, gloves, and mask. Nevertheless, my learning curve and my fondness for those women and their babies skyrocketed and I felt myself becoming a bone fide doctor with real clinical judgment. Responsibility strikes again.

The added pressure and need for such judgment became even more acute in caring for kids as I began my internship at the Children's Hospital of the University of Cincinnati. Naively, I had ranked Cincinnati Children's first solely because it was in the beloved city of my boyhood. I had no idea it was and still is one of the best pediatric facilities in the world. Given my zero perspective of academic medicine at the time, I am shocked, but grateful they took me. I had to be at the bottom of their rank list. On July 1, I began my ‘baptism by fire’. I had already survived an on-call schedule of 36-on, 12-off on the eastern shore of Virginia and in the delivery suite at MCV, but that was for a month. Here I was to spend every other night, largely awake, in the bowels of the hospital for 8 months of the year. The schedule for the junior assistant resident and senior assistant resident were the same as mine and the chief resident was on-call every night – God help him. My attendings had done the same and genuinely believed that the main disadvantage of every-other-night call was that one missed the opportunity to learn on half the patients. ‘Welcome to real doctoring!’, I thought.

A former camp counselor, I loved the kids, but nostalgically I remembered being very happy taking care of the old vets at the McGuire VA too. These heroes of another era were so appreciative of even the smallest kindness a doctor could show them. As a consequence, in starting out my career at Cincinnati Children's, my intention was to become board-certified in both pediatrics and internal medicine. There was no Med-Peds residency as yet and no time to reminisce or think about that. I was strapped to a wooden plank heading for a buzz saw in this internship. I never met the hospital operator, but I grew to hate the poor woman. She was probably a very sweet person with a pretty home and a nice family. Her voice on the overhead speakers was not at all strident. Indeed, remembering it in a detached, objective way, it was rather delicate and soothing. But every other night, seemingly all night long, the halls resounded incessantly with, ‘Doctor Fisher, call 293’ (the extension number for the emergency room). Each overhead page meant another child was to be admitted to my service. One fateful night of call, in the wee small hours of the morning I was in the midst of evaluating the 12 sick kids (including one with diabetic ketoacidosis) already admitted to me, when her dulcet page portended the admission of a second child with ketoacidosis. I ducked into an alcove somewhere and privately cried for a moment until I could catch my second wind. The head nurse, Ms. Pauline Heymann, a kind, stately, sixtyish woman in her crisp, white uniform and cap and white oxfords spotted me in my despair and patted my shoulder gently and understandingly. Her words of encouragement are long forgotten, but I thank her for helping me survive that night. Responsibility this time was ramming my clinical education down my throat – with a cruel, rigid scope. My supervisors were empathetic and supportive. They really did have my back, but they expected me to ‘man up’, make careful decisions, and take good care of my patients. For example, if one of my admissions had been a patient of one of our hematology attendings and I hadn't personally reviewed the patient's Wright-stained blood film, he would chew me up and spit me out on morning rounds. He was right, but responsibility had jagged teeth at times. I still treasure all those wonderful kids and that world-class faculty, but I abandoned the idea of being double-boarded, left the kids behind, and switched to internal medicine the next year.

Resident call on the UC medical service was every third night. My fellow house staff whined about the frequency, but I thought I was on vacation! Now for the first time, I was in charge of the team. Our unspoken commitment was to protect our attending, let him (in those patriarchal days) teach 3 days a week, and call only when we truly did not know what to do after rifling through the pages of Harrison's or Cecil's textbooks of internal medicine. One call night I was feeling pretty perspicacious as I made the diagnosis of Addison's disease in a tan-skinned, wasted, hypotensive ex-soldier with hyponatremia and hyperkalemia who presented to the VA emergency room. Unfortunately, I had carelessly glossed over his history of rheumatic fever as a child and because of his classic ‘small, quiet heart’ hadn't heard any murmur on exam. I found a recent article on treatment and began fluids and hydrocortisone without calling our attending, fully confident to receive the accolades due to me on rounds the next day. Having read up on the disease, I was ‘loaded for bear’ and delivered a rather theatrical presentation the next morning. My attending politely listened, examined the patient, and then questioned me as to why he had the early signs of congestive heart failure with an S3, a loud murmur of mitral regurgitation, and bibasilar rales. Red-faced, I quickly realized that with my steroids and saline I had volume-expanded a patient with significant valvular heart disease. Fortunately, my patient fared well in spite of me. Tinsley Harrison I was not, but I was still learning by having been given responsibilities and the successes and failures which go with it.

My residency was interrupted by a stint in the US Navy as a Flight Surgeon, countless flight physicals, and the occasional patients whom I medicated or sutured when on-call, but I returned 3 years later to finish my medicine residency. The faces of the interns and students had gotten younger, but my routine and the tasks were the same – work up patients and write notes (WP), teach the younger folks (T), read (R), make work rounds (WR) and attending rounds (AR), and go to conferences (C). I didn't fully understand academic medicine until I began my fellowship in infectious diseases which required scholarly output for the first time as well as quality patient care. Once again, we ID fellows ran the consult service with cameo appearances by the attendings who were more focused on their research and endorsed our notes with a quick slash of the pen. I hadn't changed my stripes an iota. It was still – WP, T, R, WR, AR, and C.

Detailed faculty documentation requirements began in earnest with the birth of diagnosis-related groups (DRGs) (1) at about the same time I arrived at the Medical College of Georgia (of Augusta University) as a green assistant professor. I was expecting to follow in the footsteps of my attendings-of-old – teaching young people, affixing my surname on progress notes, writing papers, and letting the residents run the service like I had. Instead, billing patients at the right level of care rather abruptly became my most important function. The federal government now demanded more written visibility from me – longer notes, requisite numbers of responses of the patients to my review of their organ systems, and proof of body parts examined – or it wouldn't pay my hospital. Insurance companies soon followed the lead of the Feds. Resident, intern, and student notes became inconsequential. At one point in our hospital, attending physicians were required to hand-write a complete history and physical examination on each new admission and re-iterate salient features of patients’ daily progress. I found myself ‘back in the saddle’ of my residency again. It wasn't long before I began receiving passive–aggressive communications about missing components of my notes from chart reviewers employed by our billing office. Each of these oversights had to be fixed before discharge. Because others had begun their notes one line below mine – the one with the missing elements – I developed a new talent at cramming legibly in the margin and, with up- or down-arrows, between lines. Moreover, despite my lack of ability and interest in the business of medicine, I was given an assignment that none of my medical training had prepared me for: billing my patients for each encounter at the correct Current Procedural Terminology (CPT) code of the Healthcare Common Procedure Coding System (HCPCS) (2) – checking boxes on a sheet in each chart labeled 99221-3 or 99231-3. Identifying the date and the level of care I rendered, the hospital billed patients accordingly in my name. This task couldn't be done during or immediately after morning rounds with the residents and students because of noon conferences. Under pain of excommunication, I had to find time to do it sometime that afternoon. Naturally my time-window frequently did not correspond to whether the patients and their charts were on the ward or off to some diagnostic or therapeutic procedure or already discharged. As one might anticipate, many billing opportunities were missed in this system and led to more dunning, delinquency messages. I doubt if I was alone in this struggle and sensed that, unlike my revered predecessors in academic medicine, attending physicians were fast becoming the enemy of hospital administrators and chairs of departments.

The electronic medical record has solved the cramming problem with a mere keystroke for us attendings once we become facile at logging on, finding the offending note, and adding the required *ad valorem* language. However, notification of a delinquency often occurs days to weeks after the patient encounter making the truthfulness of these mercenary addenda dependent on remote recall. Curiously, many of the ones I have read written by other faculty are word-for-word like previous entries.

I hesitate to use that hackneyed ‘p’ word, but there has been an unequivocal paradigm shift in academic medicine today. Fear of litigation and multi-million-dollar settlements resulting from medical errors by trainees is undoubtedly one of the driving forces behind the shift. Nevertheless, most teaching hospital CEOs will cite patient safety and outcomes for strictly enforcing the Accreditation Council for Graduate Medical Education's guidelines for reduced work hours and curtailed autonomy for residents. This is despite the fact that definitive data showing increased mortality for traditional models of patient care and graded, increasing responsibility for trainees are lacking. Indeed, the recent Study to Understand Nighttime Staffing Effectiveness in a Tertiary ICU (SUNSET-ICU) actually showed that in-house residents with access to fellows and attendings by phone produced the same outcomes as staffing the ICU with board-certified, critical care specialists supervising residents at night (3). Increased supervision and its unproven effect on patient safety should be studied to determine if this is the most effective and efficient way to improve outcomes (4).

Attending physicians in many academic medical centers are writing fewer papers and in real danger of ‘burnout’ from an increasing workload and decreasing job satisfaction (5). They truly have become the ‘fall guys’ (and gals) with all the responsibility. The young ones are getting their first taste of it immediately *after* they complete their specialty training rather than from the get-go and it will at long last begin to teach them as it taught me. Unfortunately, it will teach them less about taking care of patients. Those of us who are ‘long-in-the-tooth’ have been residents since the first day we appeared on the wards.

## References

[1] Russell GI, Alexander BD (2011). Terminology. Fundamentals of health law 1.

[2] American Medical Association Current procedural terminology.

[3] Kerlin MP, Small DS, Cooney E, Fuchs BD, Bellini LM, Mikkelsen ME (2013). A randomized trial of nighttime physician staffing in an intensive care unit. N Engl J Med.

[4] Halpern SD, Detsky AS (2014). Graded autonomy in medical education – Managing things that go bump in the night. N Engl J Med.

[5] Shanafelt TD, West CP, Sloan JA, Novotny PJ, Poland GA, Menaker R (2009). Career fit and burnout among academic faculty. Arch Intern Med.

